# The Relationship between Fatigue and Actigraphy-Derived Sleep and Rest–Activity Patterns in Cancer Survivors

**DOI:** 10.3390/curroncol28020113

**Published:** 2021-03-10

**Authors:** Tristan Martin, Rosie Twomey, Mary E. Medysky, John Temesi, S. Nicole Culos-Reed, Guillaume Y. Millet

**Affiliations:** 1 Faculty of Kinesiology, University of Calgary, Calgary, AB T2N 1N4, Canada; tristan.martin@unicaen.fr (T.M.); rosie.twomey@ucalgary.ca (R.T.); medysky@ohsu.edu (M.E.M.); john.temesi@northumbria.ac.uk (J.T.); nculosre@ucalgary.ca (S.N.C.-R.); 2 UMR-S 1075 COMETE: MOBILITES “Vieillissement, Pathologies, Santé”, INSERM, Normandy University, 14032 Caen, France; 3 Ohlson Research Initiative, Arnie Charbonneau Research Institute, Cumming School of Medicine, University of Calgary, Calgary, AB T2N 4Z6, Canada; 4 School of Nursing, Oregon Health and Science University, Portland, OR 97239, USA; 5 Faculty of Health & Life Sciences, Northumbria University, Newcastle upon Tyne NE1 8ST, UK; 6 Department of Oncology, Cumming School of Medicine, Calgary, T2N 4N1, Canada; 7 Department of Psychosocial Resources, Tom Baker Cancer Centre, Alberta Health Services, Calgary, AB T2N 4N2, Canada; 8 Univ Lyon, UJM Saint-Etienne, Inter-University Laboratory of Human Movement Biology, EA 7424, 42023 Saint-Etienne, France

**Keywords:** cancer-related fatigue, actigraphy, rest–activity cycle, insomnia

## Abstract

Cancer-related fatigue can continue long after curative cancer treatment. The aim of this study was to investigate sleep and rest–activity cycles in fatigued and non-fatigued cancer survivors. We hypothesized that sleep and rest–activity cycles would be more disturbed in people experiencing clinically-relevant fatigue, and that objective measures of sleep would be associated with the severity of fatigue in cancer survivors. Cancer survivors (*n* = 87) completed a 14-day wrist actigraphy measurement to estimate their sleep and rest–activity cycles. Fatigue was measured using the Functional Assessment of Chronic Illness Therapy Fatigue Scale (FACIT-F). Participants were dichotomised into two groups using a previously validated score (fatigued *n* = 51 and non-fatigued *n* = 36). The participant’s perception of sleep was measured using the Insomnia Severity Index (ISI). FACIT-F score was correlated with wake after sleep onset (*r* = −0.28; *p* = 0.010), sleep efficiency (*r* = 0.26; *p* = 0.016), sleep onset latency (*r* = −0.31; *p* = 0.044) and Insomnia Severity Index (ISI) score (*r* = −0.56; *p* < 0.001). The relative amplitude of the rest–activity cycles was lower in the fatigued vs. the non-fatigued group (*p* = 0.017; *d* = 0.58). After treatment for cancer, the severity of cancer-related fatigue is correlated with specific objective measures of sleep, and there is evidence of rest–activity cycle disruption in people experiencing clinically-relevant fatigue.

## 1. Introduction

Cancer-related fatigue (CRF) is a common and debilitating symptom experienced by cancer survivors [1]. It is not relieved by rest or sleep and has been described as a severe and unrelenting feeling of fatigue, or a sustained sense of exhaustion, interfering with usual function, and it therefore can negatively impact overall quality of life [2]. CRF can develop during or after cancer treatment, and for approximately one-third of people, it can persist for over 5 years [3]. The potential mechanisms underlying the onset and persistence of CRF are complex and include alterations in muscle metabolism, cytokine dysregulation, hypothalamic–pituitary–adrenal axis disruption and circadian rhythm dysregulation [4,5]. Disruption of the sleep–wake cycle may contribute to CRF, and CRF often co-occurs with chronic sleep disturbance as part of a multi-symptom cluster [6].

Patient-reported outcome and several psychometric tools are available for the screening and assessment of CRF [7,8]. The Functional Assessment of Chronic Illness Therapy Fatigue Scale (FACIT-F; [9]) is widely used for the assessment of CRF severity, with a proposed cut-off point for the diagnosis of CRF [10]. There are no objective measures of CRF, though physiological correlates of CRF are under investigation [11]. The phenomenon is best understood as a subjective awareness (described more broadly in neurological illnesses as “perception of fatigue”) [12]. Although psychometric tools measuring perceptions of sleep exist, objective measures of sleep are well established. The gold standard for the objective measurement of sleep is laboratory-based polysomnography.

A more practical alternative is wrist actigraphy, which has become a key assessment tool in sleep research [13]. Actigraphy is non-intrusive, can be used to estimate sleep in the participant’s habitual sleeping environment and permits continuous monitoring of both sleep and activity over long periods in many participants. Because some cancer survivors experience cognitive impairment after cancer treatment, a more objective measure of sleep may be desirable [14].

Poor sleep is commonly reported in cancer survivors [6]. The overwhelming majority of studies measuring sleep during and after cancer treatment use no objective measurement techniques [15]. A robust finding is that there is a discrepancy between subjective and objective measures of sleep in people with a sleep disorder [16]. Many people with insomnia overestimate the time taken to fall asleep and underestimate the total amount of sleep relative to objective measures [16], and such discrepancies have also been reported in cancer survivors [17]. Many of the studies investigating CRF and sleep have only used subjective measures of sleep disruption, and a low to moderate correlation has been reported for CRF and subjective sleep quality in survivors of breast cancer [18]. The relationship with objective measures has received less attention, particularly after cancer treatment where CRF may persist indefinitely for some people. In one example, increased fatigue in association with treatment for breast cancer was associated with disrupted circadian activity rhythms [19]. Overall, the literature suggests that CRF may be related to sleep and rest–activity cycle disturbances [20].

The relationship between perceived fatigue and sleep and rest–activity patterns in cancer survivors post-treatment has not been fully elucidated. Therefore, the primary aim of this study was to investigate objectively-estimated sleep and rest–activity cycles in fatigued and non-fatigued cancer survivors. We hypothesized that sleep would be more disturbed in people experiencing clinically-relevant CRF, and that objective measures of sleep would be associated with the severity of fatigue in cancer survivors. A secondary aim was to investigate the mismatch between subjective and objective measures of sleep in a sub-set of our participants.

## 2. Material and Methods

This work was performed at the Human Performance Laboratory, Faculty of Kinesiology, University of Calgary, Calgary, Canada.

### 2.1. Participants

Participants were recruited via the Alberta Cancer Registry (Alberta Health Services, Canada). Data extraction criteria included age (≥18 and ≤75 years), a diagnosis of any invasive cancer and postal codes within 20 km of the University of Calgary. From the resulting extraction, equal numbers of males and females were randomly sampled and sent a confidential invitation letter from the registry (such that the research team did not know who received the invitation, but participants could then contact the research team if interested). Participants were also recruited via liaising with clinicians and/or advertising at cancer centres local to the University of Calgary. Participants were eligible if they had completed initial cancer treatment and were not scheduled to receive further treatment during the study period. Initially, 57 participants were recruited for the study (REB14-0398: FACIT-F > 34, *n* = 36; FACIT-F ≤ 34, *n* = 20). One shift worker was not included in the final data analysis. The study was later extended to include an exercise program for people with clinically-relevant CRF i.e., FACIT-F ≤ 34, *n* = 31 (HREBA.CC-16-10-10, see also Twomey et al. [11]), meaning that, in total, 87 cancer survivors (53 females) provided written informed consent to participate in and complete the study procedures. The characteristics of the participants are presented in Table 1. Approval for all procedures was obtained by the Conjoint Health Research Ethics Board and the Health Research Ethics Board of Alberta Cancer Committee (REB14-0398 and HREBA.CC-16-10-10, respectively).

### 2.2. Study Design

Participants attended two lab visits. On the initial lab visit, participants completed a health screening and a number of other procedures as part of a wider project investigating cancer-related fatigue. Participants were provided with an actigraphy system and a sleep diary (see later Sections) and the data collection period was set to 2 weeks. After the data collection period, participants attended the lab to return the MotionWatch 8 and the sleep diary. On this lab visit, participants completed the FACIT-F scale and the Insomnia Severity Index (ISI) [21].

### 2.3. Actigraphy

The MotionWatch 8 actigraphy system (CamNtech Ltd., Fenstanton, Cambridgeshire, UK) is an unobtrusive, waterproof, wrist-worn device containing a light sensor and a tri-axial accelerometer detecting acceleration in a 0.01–8 g range. The device recorded 30-s epochs (as per manufacturer guidelines) and light intensity. The device was worn on the non-dominant wrist for a continuous 2-week period to obtain aggregated measures that characterize individuals and quantify sleep in accordance with established recommendations [13,22].

### 2.4. Sleep Diary

Participants were also provided with a sleep diary to complete alongside the actigraphy measurement [23] to assist with the editing of the actigraph data for better accuracy [24]. Information provided by the participants (which was self-reported) included “lights out” time (i.e., 8:30 PM), estimated sleep onset (time) and night-time awakenings (number/night).

### 2.5. Cancer-Related Fatigue

The FACT-F is a 13-item, 5-point Likert scale. The scale has a cut-off to distinguish fatigued patients from non-fatigued patients, with scores ≤34 indicating clinically relevant fatigue [10]. The scale has high internal consistency [9] and is widely used in the literature [25].

### 2.6. Insomnia Severity

The perceived severity of insomnia over the previous 2 weeks was measured using the ISI [21]. Questions addressed difficulty falling asleep, the maintaining of sleep and the frequency of early morning awakenings, as well as the degree of dissatisfaction with current sleep. The ISI has been validated in cancer survivors [26]. Items are rated from 0 (none) to 4 (very severe), scores ranged from 0–28 (≥8 indicates clinical insomnia) and the list gave maximal sensitivity and specificity for the detection of sleep difficulties [27]. The ISI was a late addition to the protocol and, therefore, only 66 participants completed this questionnaire.

### 2.7. Data Analysis

Estimated sleep characteristics were derived from actigraphy. Actigraph data were analysed with Motionware version 1.1.20 software (CamNtech Ltd., Fenstanton, Cambridgeshire, UK). As recommended in the literature [28], actigraph data were edited to correspond with the completed sleep diary. In cases when the actigraph data and the sleep diary disagreed on the initiation of sleep, the light sensor data were used to determine the approximate “lights out” time, or when the participant first tried falling asleep. Similarly, the light data and the first large increase in activity onset were used to determine the end of the sleep window where the actigraph data and sleep diary did not correspond. This type of data editing has been performed in a previous study of older adults [24]. Sleep variables estimated by Motionware included total sleep time (TST: time spent asleep at night), sleep efficiency (SE: percentage of time spent sleeping in relation to time spent in bed), sleep onset latency (SOL: amount of time taken to fall asleep), wake after sleep onset (WASO: minutes awake after an extended period of sleep) and fragmentation index (FI: indication of sleep quality based on the sum of the “Mobile time (%)” and the “Immobile bouts ≤ 1min (%)”, as calculated by the software). A high sensitivity setting was used, with a time awake threshold of 20 counts per epoch, and sleep onset was defined as the first period with a minimum of 10 min of consecutively recorded immobile data with no more than 1 epoch of movement within that time (as validated by the manufacturer).

The characteristics of the rest–activity cycle were analysed with the Non-Parametric Circadian Rhythm Analysis (NPCRA) function. We considered the following parameters to characterize the rest–activity cycle: estimated peak time of activity (the time of day of the the peak of the cosine function fitted to the average of the 24 h data) and the relative amplitude of the rest–activity cycle (the difference between the mean activity in the estimated least and most active periods, with a theoretical range of 0 to 1, where higher values indicated a rhythm with higher amplitude) [29]. The following parameters were also estimated from the raw MotionWatch 8 data: the mean activity for wake and sleep periods (the mean amount of movement while out of bed and the mean amount of movement while in bed, respectively) and the index of activity for wake and sleep, calculated as the percentage of activity > 0 per epoch for wake and sleep periods, respectively [30,31].

### 2.8. Statistical Methods

An a priori sample size estimation was performed using G*Power 3 (v3.1.2–3.1.9) [32] for the relationship between FACIT-F and the objectively-estimated sleep parameters. For a correlation coefficient of *r* = 0.3 (i.e., a small effect), with α = 0.05 and a 1 − β = 0.80, the sample size required was calculated as 85. All data were analysed using IBM SPSS version 23 statistical software (IBM Corporation, Chicago, IL, USA). Data were checked for normality using the Skewness–Kurtosis test combined with a visual inspection of the histograms and the Q–Q plots. Data were also checked for homogeneity of variance using Levene’s test. To test the hypothesis that participants experiencing clinically-relevant fatigue would have poorer sleep than those who were not, participants were dichotomised into two groups using FACIT-F >34 and ≤34 (labelled as the non-fatigued group and the fatigued group, respectively) and parameters relating to sleep and the rest–activity cycle were compared using independent sample t-tests, or non-parametric Mann–Whitney tests when data were not normally distributed. Significant results were controlled for multiple comparisons using the Benjamini and Hochberg false discovery rate procedure [33,34]. This procedure compares each individual *p* value to its Benjamini–Hochberg critical value, (i/m)Q, where i is the rank, m is the total number of comparisons and Q is the chosen false discovery rate (FDR). FDR Q values usually range from 0.05 (relatively conservative) to 0.2 (liberal). Due to the exploratory nature of this study (in that participants’ daily living could not be controlled), the limitations of using actigraphy despite its demonstrated validity and reliability [13] and the importance of not missing a potentially meaningful difference, we chose an intermediate FDR of Q = 0.1. Effect sizes for pairwise comparisons were calculated as Cohen’s *d* [35,36]. Interpretation of the size of the effect was considered as *d* = 0.2 being small, *d* = 0.5 being medium and *d* = 0.8 being large [35]. The relationships between FACIT-F score and sleep and rest–activity outcomes were examined using Pearson correlation coefficients or Spearman correlation coefficients, as appropriate. The relationship between perceptions of sleep (ISI score) and sleep outcomes were also examined using Pearson correlation coefficients or Spearman correlation coefficients. The threshold for statistical significance of the Pearson and Spearman correlations coefficients was set at *p* < 0.05.

## 3. Results

Of the 87 participants, 51 cancer survivors had CRF (in the fatigued group) and 36 cancer survivors were non-fatigued, according to FACIT-F scores. Participant characteristics and FACIT-F scores are presented in Table 1. Time since treatment was not correlated with FACIT-F score (*r* = 0.136; *p* = 0.283). Many of the participants were highly educated (49%), white (89%), female (61%) and survivors of breast cancer (44%).

### 3.1. Objective Measures of Sleep

**Figure 1:**
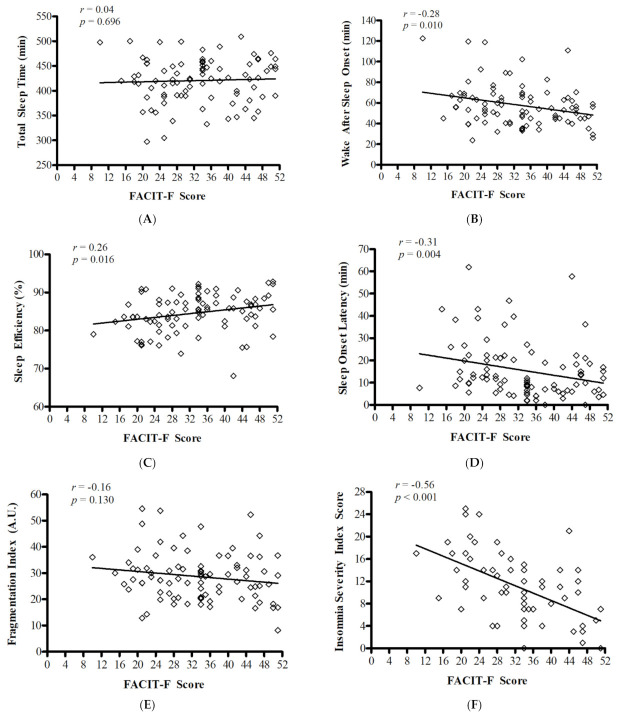
Correlations between actigraphy-derived sleep parameters and Functional Assessment of Chronic Illness Therapy–Fatigue (FACIT-F) score (panels **A**–**E**), and the correlation between the Insomnia Severity Index score and FACIT-F (panel **F**). A lower FACIT-F score indicates higher fatigue severity.

In the fatigued group, participants had greater WASO (*p* = 0.046, *d* = 0.43) and SOL (*p* = 0.053, *d* = 0.35) using the Benjamini–Hochberg procedure. No other significant differences between the groups were observed (Table 2). Overall, participants took 15 ± 13 min to fall asleep at night, were asleep for 421 ± 45 min per night (7.0 ± 0.8 h), spent 58 ± 20 min awake after initially falling asleep and had a mean SE of 84.6 ± 5.0% and an FI of 28.7 ± 9.2. FACIT-F score was correlated with wake after sleep onset (*r* = −0.28; *p* = 0.010), sleep efficiency (*r* = 0.26; *p* = 0.016) and sleep onset latency (ρ = −0.31; *p* = 0.004). FACIT-F score was not correlated with total sleep time (*r* = 0.04; *p* = 0.696) or fragmentation index (*r* = −0.16; *p* = 0.130). Correlations are presented in Figure 1A–E.

### 3.2. Rest–Activity Cycles

The amount of movement during the sleep period (mean sleep actigraphy and index of activity during sleep) were both higher in the fatigued vs. the non-fatigued group (Table 2). The relative amplitude of the rest–activity cycle was lower in the fatigued group vs. the non-fatigued group (*p* = 0.007, *d* = 0.62) using the Benjamini–Hochberg procedure. The peak time of the rest–activity cycle occurred ~40 min later in the fatigued group than in the non-fatigued group (*p* = 0.007, *d* = 0.62). The fatigued group also went to bed 25 min later (*p* = 0.03, *d* = 0.48) and woke up 40 min later (*p* = 0.009, *d* = 0.58) than the non-fatigued participants. The index of activity during the wake period (mean wake actigraphy) was not different between the groups. The FACIT-F score was negatively correlated with the peak time of the rest–activity cycle (*r* = −0.32; *p* = 0.002), bed time (*r* = −0.26; *p* = 0.014) and wake up time (*r* = −0.28; *p* = 0.008). The FACIT-F score was also correlated with the amount of movement during the sleep period (i.e., the index of activity during sleep: *r* = −0.39; *p* < 0.001), the mean sleep actigraphy (ρ = −0.39; *p* < 0.001) and relative amplitude (ρ = 0.31; *p* = 0.003). FACIT-F was not correlated with other rest–activity cycle parameters (see Supplemental Table S1).

### 3.3. Perception of Sleep Disruption

The mean ISI score was significantly higher in the fatigued group than it was in the non-fatigued group (Table 2). In the fatigued group, the vast majority of participants (83%, *n* = 35) reported clinically-relevant sleep disruption (ISI total score ≥ 8 [27]). Of these 35 participants, 20 reported sub-threshold insomnia (ISI total score 8–14), 12 reported insomnia of moderate severity (ISI total score 15–21) and 3 reported severe insomnia (ISI total score 22–28). In the non-fatigued group, half of the participants (50%, *n* = 12) reported clinically-relevant sleep disruption (ISI total score ≥ 8) [27]. Of these 12 participants, 10 reported sub-threshold insomnia (ISI total score 8–14) and 2 reported insomnia of moderate severity (ISI total score 15–21).

The relationship between perceived sleep and perceived fatigue is presented in Figure 1F. The total ISI score was negatively and significantly associated with the FACIT-F score (*r* = −0.56; *p* < 0.001). That is to say, more severe perceived difficulties with sleep were associated with more severe ratings of fatigue. We also examined the relationships between total ISI score and actigraphy-estimated sleep outcomes. The total ISI score was not correlated with TST (*r* = −0.076; *p* = 0.546), WASO (*r* = 0.24; *p* = 0.051) or FI (*r* = 0.06; *p* = 0.608). However, the total ISI score was significantly correlated with SE (*r* = −0.34; *p* = 0.006) and sleep onset latency (ρ = 0.30; *p* = 0.013) (see Supplemental Table S2).

## 4. Discussion

The primary aim of this study was to investigate objectively-estimated sleep and rest–activity cycles in fatigued and non-fatigued cancer survivors. In line with our hypothesis, some actigraphy-derived markers of sleep were associated with the severity of fatigue in cancer survivors, including parameters associated with more periods of awakening and movement during the sleep period. To our knowledge, this is the first study to investigate sleep and rest–activity cycle disruption months and years after cancer treatment, comparing fatigued and non-fatigued cancer survivors. A secondary aim of this study was to investigate the association between subjective and objective measures of sleep in a sub-set of our participants. We found that people with CRF perceived their sleep as worse than that of non-fatigued survivors, and this perception was associated with both fatigue severity and several (although not all) objective sleep parameters.

### 4.1. The Relationship between Fatigue and Actigraphy-Derived Sleep and Comparison to Reference Data for Sleep Duration and Quality

CRF is a complex issue that, for some people, can continue for years after cancer treatment. CRF and poor sleep can occur simultaneously, but the relationship between CRF and actigraphy-derived estimates of sleep has not been investigated after cancer treatment. The current findings show a small but significant association between fatigue assessed with FACIT-F and specific objective parameters, including wake after sleep onset, sleep efficiency and sleep onset latency. In cancer survivors, difficulties initiating sleep and the robustness/quality of the sleep episode may contribute to the perception of CRF which persists after treatment completion. This is a major concern for health-related quality of life and societal issues, including return to work and productivity at work [37]. Interestingly, there was no relationship between CRF and total sleep time. Existing research shows that the time devoted to sleep is similar for cancer patients and healthy controls [38]. Moreover, total sleep time has been shown to be similar at across various points during cancer treatment, and similar to that of a healthy population up to 1 year after treatment [39].

Using dichotomized groups based on a pre-specified and validated cut off point [10], we found that participants categorized as fatigued vs. non-fatigued had a marginally greater time spent awake after sleep onset and time to fall asleep. The current study did not include a control group, but recent data from the National Sleep Foundation may be used as reference data for sleep duration and quality. We identified 38 people (44%) who slept less than the recommended sleep duration [40]. These participants were approximately evenly distributed across the dichotomised groups (43% of the fatigued group and 44% of the non-fatigued group, respectively). Concerning other sleep parameters, a good sleep quality was defined as WASO < 51 min, SOL < 30 min or SE > 85% [41]. Specifically, only 37% of fatigued people were awake at night for < 51 min, versus 50% of people in the non-fatigued group. There was also a small and small–medium effect for sleep efficiency and sleep onset latency, respectively. The poor quality of sleep in cancer survivors has been mainly associated with a high wake time at night and a long sleep onset latency, rather than a lesser total nocturnal sleep time [42]. The fact that approximately double the number of participants had issues with sleep efficiency and latency in the fatigued vs. non-fatigued group suggests that difficulties with beginning/maintaining sleep (like in insomnia) relate to subjective feelings of fatigue. Specifically, for latency, 16% of the fatigued group took longer than 30 min to fall asleep (8% of the non-fatigued group) and 59% had a sleep efficiency of less than 85% (versus 33% of the non-fatigued group). A low sleep efficiency is a major concern, given that a less efficient sleep has been associated with lower survival in people living with and beyond cancer [43]. In summary, more severe fatigue seems to be related with a higher difficulty in achieving a good sleep. Even though the precise mechanisms are unknown, both CRF and sleep disorders share a common symptom cluster (including the modification of immune function, dysregulation of the hypothalamic–pituitary axis and circadian rhythms, pain and depression) that may be the basis of a reciprocal relationship between sleep disorders and CRF [5,6]. Further investigation is required, since cancer survivors in the present study had completed initial cancer treatment only (e.g., surgery, chemotherapy and/or radiation therapy). Some long-term adjuvant treatments, specifically hormonal therapy, may affect daytime activity and sleep quality [44].

### 4.2. The Relationship between Fatigue and Rest–Activity Cycles in Cancer Survivors

Sleep has a reciprocal relationship with circadian rhythm disturbances in that sleep disruption might adversely affect circadian function, and disrupted circadian function can contribute to the development of sleep disturbances [43]. The estimated cosine peak time of the rest–activity cycle occurred later in fatigued survivors than in non-fatigued survivors in our study, which was correlated with CRF score. Fatigued survivors also had delayed bed and wake up times. Phase delays of the rest–activity cycle have been previously observed in patients with depressive symptoms presenting low sleep quality [45]. More importantly, despite similar indices of daytime activity, fatigued survivors exhibited more body movement during sleep and a lower relative amplitude of the rest–activity cycle cosine estimation. Both were also associated with CRF. A lower amplitude of the rest–activity cycle has previously been observed after treatment in cancer survivors compared to control participants [19]. Previous results in cancer survivors have highlighted a clear dichotomy between day-time and night-time actigraphy, with high activity during wake time and low activity during night-time being associated with better quality of life [46]. Interestingly, Ancoli–Israel et al. [39] observed that characteristics of the rest–activity cycle returned to baseline one year after treatment, despite being lower than those of healthy controls. Although no comparison to a control population was included in the current study, our results show that fatigued participants have a lower amplitude of the rest–activity cycle than do non-fatigued participant months and years after treatment. An altered rest–activity cycle may also be related to other symptoms associated with cancer, such as pain and depression [6]. Further experiments in people with clinically-relevant CRF are needed to better understand the link between circadian rhythm disruption and persistent CRF after treatment.

### 4.3. The Mismatch between Subjective and Objective Measures of Sleep

Subjective sleep does not always correspond with actigraph data due to the presence of other symptoms or psychological and physiological factors [38]. However, fatigue is one of the most reported complaints of patients with insomnia [47]. The longer time spent awake during the night and the longer time taken to fall asleep observed in fatigued participants corroborate the higher subjective feeling of insomnia in this study. The ISI was chosen as a self-report since insomnia is common in cancer survivors who report having sleep disorders [48]. We found that the majority (83%) of fatigued participants in the present study reported significant sleep disruption (ISI total score ≥ 8), whereas this was reduced to 50% in the non-fatigued group. Dirksen et al. [49] showed that cancer survivors with the highest level of fatigue reported higher scores on the ISI, which was in accordance with the present study, suggesting that CRF may be linked to the perception of sleep disturbance in cancer populations. ISI scores in our study were lower (less self-reported insomnia) than in previous studies of cancer survivors where ISI was used to assess insomnia after treatment [50]; however, these studies focused on cancer survivors with clinical insomnia.

Based on the results of the present study, interventions for CRF after cancer treatment should target both the perception of sleep and the improvement of objectively-measured sleep. Two interventions that may have promise for treating sleep disturbance are exercise [20] and cognitive behavioural therapy [51].

## 5. Limitations

There are several limitations of the current study. As previously highlighted, these results are based on a sample of predominantly white, female survivors of breast cancer and therefore may not be representative of a more diverse population or generalizable to specific tumour groups. Dichotomization of the FACIT-F score, although using a validated cut-off [10], may have resulted in the misclassification of some individuals. We did not ask participants about the perception of their sleep quality and quantity prior to their cancer diagnosis because the recall period would have been >12 months for most. Therefore, it was not possible to comment on whether sleep and rest–activity cycle disruption was cancer-related. Future longitudinal studies should aim to investigate the relationship between fatigue and sleep during the period between diagnosis and treatment, and at time-points encompassing treatment and recovery. Actigraphy has some key limitations that have been discussed previously [13], such as the underestimation of SOL and the overestimation of SE. It cannot be considered as a substitute for clinical interviews or overnight polysomnography, and information about the modification of sleep architecture between people with and without CRF must also be measured in future studies. There is also a risk of bias due to lack of compliancy in participants completing the sleep diary (which was used to edit the actigraph data to improve accuracy). The limitation of paper diaries is that aspects of compliance (such as the recall period) cannot be monitored by the experimenter in real time, and participants may not remember to complete the diary every day [52]. However, this limitation was minimized by using the light sensor data to indicate sleep and wake onset when the sleep diary and actigraph data were otherwise incongruent [52].

## 6. Conclusions

This is the first study to show that after treatment for cancer, the severity of CRF is correlated with specific objective measures of sleep (including sleep onset latency, wake time at night and sleep efficiency), but not total sleep duration. The subjective perception of difficulties with sleep was worse in people with clinically-relevant CRF. In addition, the severity of CRF is correlated to the amount of movement during the sleep period, and we observed evidence of disrupted rest–activity cycles in people experiencing CRF. Future studies should focus on the potential benefits of interventions that influence both objective and subjective measure of sleep in people with CRF after cancer treatment, particularly exercise. These findings have direct consequences on the rehabilitation process, even months and years after cancer treatment.

## Figures and Tables

**Table 1:** The characteristics of the participants.

		All	Fatigued	Non-Fatigued
*N*		87	51	36
**Age (years)**		55.8 ± 10.2	54.2 ± 8.9	58.2 ± 11.6
**Months Since Treatment**		33.4 ± 28.7	40.1 ± 28.9	29.9 ± 27.1
**Sex**	Female	53 (61)	32 (63)	21 (58)
	Male	34 (39)	19 (37)	15 (42)
**Ethnicity (self-identified) ^a^**	White	76 (89)	43 (88)	33 (98)
	Middle Eastern	1 (1)	0 (0)	1 (3)
	Asian	5 (6)	4 (8)	1 (3)
	Black	1 (1)	1 (2)	0 (0)
	First Nations	2 (2)	1 (2)	1 (3)
**Marital Status ^a^**	Married/Common Law	64 (75)	34 (69)	30 (83)
	Divorced	12 (14)	6 (12)	6 (17)
	Single	7 (8)	7 (14)	0 (0)
	Widowed	2 (2)	2 (4)	0 (0)
**Education ^a^**	University	42 (49)	25 (51)	17 (48)
	College	26 (31)	16 (33)	10 (28)
	Secondary School	14 (17)	5 (10)	9 (25)
	Other	3 (4)	3 (6)	0 (0)
**Employment Status**	Part-Time	14 (17)	6 (12)	8 (22)
	Full-Time	34 (40)	23 (47)	11 (31)
	Retired	20 (24)	6 (12)	14 (39)
	Unemployed	7 (8)	5 (10)	2 (6)
	Disability/Leave	10 (12)	9 (18)	1 (3)
**Cancer Type**	Breast	38 (44)	22 (43)	16 (44)
	Prostate	15 (17)	4 (8)	11 (31)
	Head & Neck	8 (9)	6 (12)	2 (6)
	Colorectal	7 (8)	5 (10)	2 (6)
	Hematologic	1 (1)	1 (2)	0 (0)
	Other	18 (21)	13 (26)	5 (14)
**FACIT-F Score**		33 ± 10	26 ± 6	43 ± 6

^a^ Demographic data about ethnicity, marital status and education were not provided for two fatigued participants. Data are presented as mean ± SD for continuous data or count (percentage) for categorical data. Some values may not add up to 100% due to rounding. Other cancer types included bladder, testicular, endometrial, lymphoma, kidney, lymphoma, papillary, oesophageal, thyroid, seminoma, choriocarcinoma, pancreatic, brain and cervical.

**Table 2:** Sleep and rest–activity cycle parameters for fatigued and non-fatigued cancer survivors.

	Fatigued (*n* = 51)		Non-Fatigued (*n* = 36)			
Outcome	Mean	SD	Mean	SD	*P* (*q*)	Effect Size
Sleep (Actigraphy)						
Total Sleep Time (min)	422.6	44.9	417.9	46.3	0.632 (0.100)	0.10
Sleep Efficiency (%)	84.1	4.7	85.3	5.3	0.285 (0.071)	0.23
Sleep Onset Latency (min) ^np^	17.3	13.1	12.9	11.9	0.053 ^a^ (0.064)	0.35
Wake After Sleep Onset (min)	61.2	22.4	52.9	15.7	0.046 ^a^ (0.057)	0.43
Fragmentation Index	29.5	9.4	27.5	8.9	0.305 (0.086)	0.23
Perceptions of Sleep						
Insomnia Severity Index	12.7	5.6	8.0	5.6	0.020 ^a^ (0.043)	0.84
Rest–activity Cycle						
Relative amplitude ^np^	0.88	0.07	0.91	0.05	0.017 ^a^ (0.029)	0.58
Bed times	23 h 21	0 h 57	22 h 55	00 h 48	0.033 ^a^ (0.05)	0.48
Wake up times	7 h 40	01 h 07	6 h 59	01 h 11	0.009 ^a^ (0.021)	0.58
Peak Time (hh:mm)	14 h 40	01 h 14	14 h 00	00 h 53	0.007 ^a^ (0.007)	0.62
Mean Sleep Actigraphy (MW8 counts) ^np^	15.6	6.9	12.8	6.5	0.018 ^a^ (0.036)	0.42
Mean Wake Actigraphy (MW8 counts)	101.3	33.6	109.6	37.0	0.289 (0.079)	0.23
Index of Activity during Wake (%)	68.9	9.3	70.8	10.0	0.364 (0.093)	0.20
Index of Activity during Sleep (%)	14.3	3.8	12.8	3.7	0.008 ^a^ (0.014)	0.60

^a^ indicates *p* values which are significant using the Benjamini–Hochberg procedure with a false discovery rate of Q = 0.1. ^np^ indicates a non-parametric Mann–Whitney test.

## Data Availability

The data presented in this study are available on request from the corresponding author.

## Supplementary Materials

The following are available online at https://www.mdpi.com/1718-7729/28/2/113/s1, Table S1: Correlation between sleep and rest-activity cycle parameters, Functional Assessment of Chronic Illness Therapy-Fatigue Scale (FACIT-F); Table S2: Correlation between sleep estimated parameters and Insomnia Severity Index (ISI).
