# Supplementary material for: The Relationship between Fatigue and Actigraphy-Derived Sleep and Rest–Activity Patterns in Cancer Survivors
Source: Curr Oncol. 2021 Mar 10;28(2):1170–82. doi: 10.3390/curroncol28020113 (PMC8025824; doi:10.3390/curroncol28020113)
Supplement: Supplementary file 1 [file curroncol-28-00113-s001.pdf]

Copyright © Multimed Inc.

CURRENT ONCOLOGY
A Canadian Cancer Research Journal

Supplemental Materials for

The Relationship between Fatigue and Actigraphy-Derived Sleep and Rest-Activity Patterns in Cancer Survivors

Tristan Martin, Rosie Twomey, Mary E. Medysky, John Temesi, S. Nicole Culos-Reed and Guillaume Y. Millet

Listing of Supplemental Material(s):

Supplemental Table 1: Correlation between sleep and rest-activity cycle parameters, Functional Assessment of Chronic Illness Therapy-Fatigue Scale (FACIT-F)

Supplemental Table 2: Correlation between sleep estimated parameters and Insomnia Severity Index (ISI).

Copyright © Multimed Inc.

Supplemental Table 1 : Correlation between sleep and rest-activity cycle parameters, Functional Assessment of Chronic Illness Therapy-Fatigue Scale (FACIT-F)

	FACIT-F		
	Pearson	Sig.	N
TST	0,042	0,696	87
WASO	-,275	0,010	87
SE	,258 [b]	0,016	87
SOL[np]	-,305 [b]	0,004	87
FI	-0,164	0,130	87
Index activity wake %	0,040	0,712	87
Index activity sleep %	-,385 [a]	0,000	87
Mean wake Actigraphy	0,092	0,396	87
Mean Sleep Actigraphy[np]	-,392 [a]	0,001	87
Amplitude (RA)[np]	,313 [a]	0,003	87
Peak time	-,323 [a]	0,002	87
IS (stability)	0,168	0,120	87
IV (variability)	0,015	0,889	87
Bed time	-,263 [b]	0,014	87
Wake-up time	-,282 [a]	0,008	87

[a] Correlation is significant at 0,01 level (bilateral).
[b] Correlation is significant at 0,05 level (bilateral).
[np] indicate non parametric spearman correlation test

Copyright © Multimed Inc.

Supplemental Table 2: Correlation between sleep estimated parameters and Insomnia Severity Index (ISI).

	ISI		
	Pearson	Sig.	N
FACIT-F	-,560[a]	0,000	66
TST	-0,076	0,546	66
WASO	0,242	0,051	66
SE	-,336 [a]	0,006	66
SOL[np]	,303 [a]	0,013	66
FI	0,064	0,608	66

[a] Correlation is significant at 0,01 level (bilateral).
[b] Correlation is significant at 0,05 level (bilateral).
[np] indicate non parametric spearman correlation test
